#  Comparative Pharmacokinetics of Levofloxacin in Healthy Volunteers and in Patients Suffering from Typhoid Fever

**Published:** 2013

**Authors:** Muhammad Usman, Muhammad Ashraf, Muhammad Imran Khokhar, Bilal Ashiq, Muhammad Irfan Masood, Shehryar Afzal, Ovais Omer, Mohsin Ali, M. Imran Qadir

**Affiliations:** a *Institute of Pharmaceutical Sciences, University of Veterinary and Animal Sciences, Lahore, Pakistan. *; b *Department of Pharmacology and Toxicology, University of Veterinary and Animal Sciences, Lahore, Pakistan. *; c *Services Institute of Medical Sciences, Services Hospital, Lahore, Pakistan. *; d *Department of Pharmacology and Toxicology, University of veterinary and Animal Sciences, Lahore, Pakistan. *; e *College of Pharmacy, GC University, Faisalabad, Pakistan. *

**Keywords:** Pharmacokinetics, Levofloxacin, Typhoid fever, Volunteers, HPLC

## Abstract

The aim of this study was to characterize the effect of typhoid fever on pharmacokinetic parameters of levofloxacin (LF) and compare the pharmacokinetic parameters of the said antibiotic in healthy human volunteers and patients with typhoid fever. Total of 12 subjects were divided into two groups “A” (healthy volunteers) and “B” (typhoid patients). Single oral dose of LF 500 mg was given and 5 mL of blood was collected from each subject at 0, 0.25, 0.5, 1, 2, 3, 6, 12, 24, 36 and 72 h. Plasma concentrations of LF were measured by HPLC. Pharmacokinetic parameters were calculated from plasma concentration-time data by using MW/PHARM pharmacological analysis. In healthy volunteers, the average pharmacokinetic parameters were as C_max_ (6.79 μg/mL), T_max_ (1.84 h), T^½ ^(10.03 h), Ka (2.23 h^-1^), AUC (110.09 μgh/mL), Vd (85.84 L), Cl (4.57 L/h) and in typhoid patients were C_max _(6.90 μg/mL), T_max _(1.82 h), T^½^ (9.42 h), Ka (2.21 h^-1^), AUC (105.55 μgh/mL), Vd (64.31 L), Cl (4.75 L/h). The difference between pharmacokinetic parameters of LF in healthy human volunteers and typhoid patients was calculated by using unpaired t-test. As the p-value in case of all pharmacokinetic parameters was more than 0.05, the difference between pharmacokinetic parameters in both healthy human volunteers and typhoid patients was insignificant. It is concluded that there is no need to adjust the dose of LF in typhoid patients.

## Introduction

Levofloxacin (LF) is a group-III fluoroquinolone antibiotic equally effective against Gram-positive (G+ve) and Gram-negative (G-ve) bacteria (1). It is the levorotatory isomer of ofloxacin (2). It is used for the treatment of urinary tract infection, chronic prostatitis (3) prophylactically in preventing febrile episodes in patients with neutropenia and cancer (4) in nosocomial pneumonia, meningitis and skin infections (5). LF like other fluoroquinolones, is the drug of choice against *Salmonella enterica *serotype typhi and non-typhi which are resistant to the first line antibiotics (6). Pharmacokinetic studies for assuring the *in-vivo *efficacy, safety and performance of a drug are conducted by measuring the drug concentration in biological fluids through different HPLC methods. These HPLC methods include one-step protein precipitation extraction and single step liquid-liquid extraction followed by UV detection and solid-phase extraction followed by fluorescent detection (7). Pharmacokinetic studies are important for adjusting the proper dose of drug in patients with specific diseased conditions since diseased conditions greatly affect the Pharmacokinetics and Pharmacodynamics of certain antibiotics (8).

Typhoid is the systemic infection caused by a Gram-negative (G-ve) bacterium *Salmonella typhi *and is characterized by a continuous fever for 21-28 days with the involvement of lymphoid tissues. *Infections caused by Gram-negative bacteria are at the top causes of morbidity and mortality in critically ill patients *(9). Quinolones have potential advantage over the other antimicrobial agent used for the treatment of typhoid fever, due to their ability to penetrate the macrophages and achieving high concentration in bowel and bile Lumina (10). In typhoid infection, the lymphoid organs are involved and the liver is the largest lymphoid organ affected in typhoid, and also the major metabolizing organ. This fact may affect the pharmacokinetics of LF if given to typhoid patient which in turn may affect the decision for dose adjustment.

The aim of this study was to characterize the effect of typhoid fever on pharmacokinetic parameters of levofloxacin (LF) and compare the pharmacokinetic parameters of the said antibiotic in healthy human volunteers and patients with typhoid fever.

## Experimental

*Subjects*

This study was conducted on six healthy male volunteers and in six human patients suffering from typhoid fever. We calculated the sample size for this study by using the following formula (11):

$n=\frac{Z^{2}P(1-P)}{d^{2}}$

Here, *n *= sample size, *Z *= Z statistic for a level of confidence, *P *= expected prevalence or proportion and *d *= precision.

The patients were selected from Services Institute of Medical Sciences (SIMS), Services Hospital Lahore, Pakistan and six healthy volunteers were selected from community.

*Chemicals*

LF Standard (Kindly provided by Pacific Pharmaceutical Limited, Lahore, Pakistan) was applied in this study. Methanol was of HPLC grade, CuSO4.5H2O and L-Isoleucine.

*Instrumentation*

The chromatography was carried out using an Agilent 1100 series instrument equipped with a power supplier, an auto-sampler and a UV detector connected to a data collection system. The column used was C18, (0.5 μm particle size, 150 × 4.6 mm). The wavelength of detector was set at 330 nm. The temperature of column was maintained at 35°C. The isocratic mobile phase was copper sulfate pentahydrate (5 mM) containing (10 mM) L-isoleucine : methanol (87.5 : 12.5, v/v). The flow rate was set at 1.1 mL/min.

*Sample preparation*

Stock solution of LF (1 mg/mL) was prepared by dissolving 100 mg of LF standard in 100 mL of distilled water. Further dilutions were prepared in blank plasma.

Following dilutions were prepared:

0.5 μgmL^-1^, 1 μgmL^-1^,2 μgmL^-1^,5 μgmL^-1^,10 μgmL^-1^.

*Extraction of samples*

To an aliquot of plasma (1 mL) 1.5 mL of acetonitrile was added. Each sample was vortex mixed for 5 min and centrifuged at 1650 RPM for 5 min. Mixture was then stored at - 20°C for 30 min. The upper organic layer was separated with the help of pipette and was evaporated in incubator at 37°C. After the evaporation, each sample was reconstituted in 0.5 mL of mobile phase. An aliquot (20 μL) was injected into the HPLC, as shown in Illustration 1.

*Sample collection*

Time and method for sample collection were followed as used by Benko *et al., *2007 (12). LF 500 mg tablet was administered to each individual orally. Blood samples (5 mL each) were collected in heparinized test tubes at 0, 0.25, 0.5, 1, 2, 3, 6, 12, 24, 36 and 72 h from vein of either arm through a 5 CC disposable syringe of 23G needle. Plasma was separated from the samples by centrifugation at 5000 RPM for 5 min. The plasma samples were stored at - 80°C till analysis.

*Calculations*

Concentrations of LF in plasma were measured by HPLC method. Pharmacokinetic parameters were calculated by applying the plasma concentration-time data in the well known software MW/PHARM pharmacological analysis version 3.02. The bioavailability of LF after the oral administration was considered as one.

## Results

**Figure 1:**
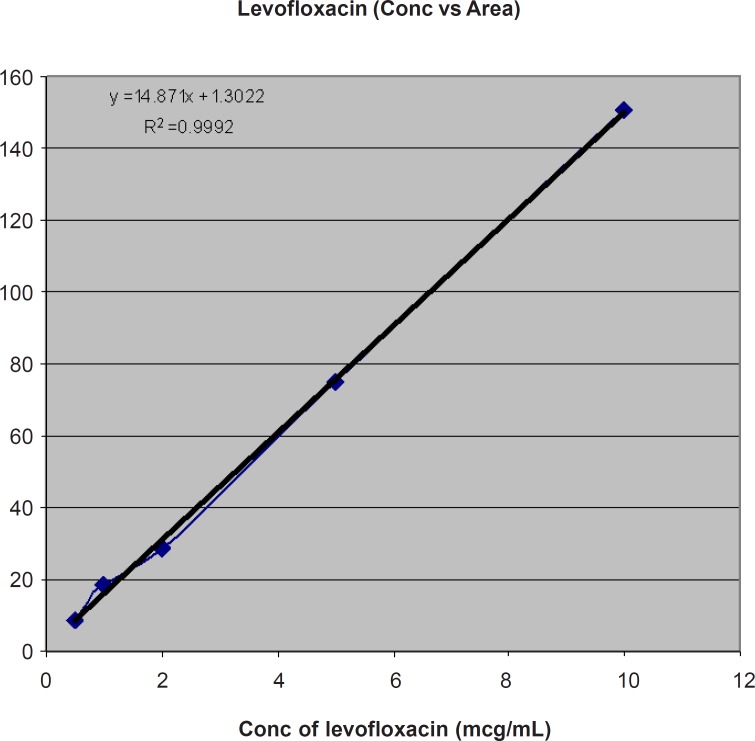
Calibration Standard curve of LF at different concentrations (μg/mL) of VS Area (MAU).

Calibration Standard curve of LF at different concentrations (μg/mL) of VS Area (MAU) is shown in Table 1 and Figure 1. 

**Table 1:** Calibration Standards of LF at different concentrations (μg/mL).

**Conc. Of LF (μg/m**L)	**Area (MAU)**
10	150.6051
5	74.8707
2	28.9171
1	18.3521
0.5	8.8784

**Figure 2:**
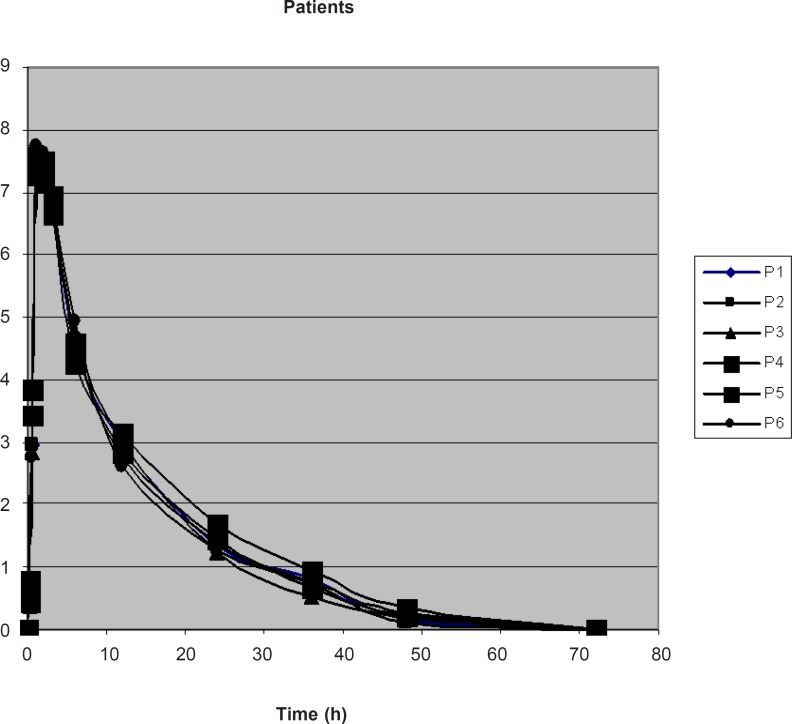
Plasma Conc. vs. Time graph of LF in 6 typhoid patients

**Figure 3:**
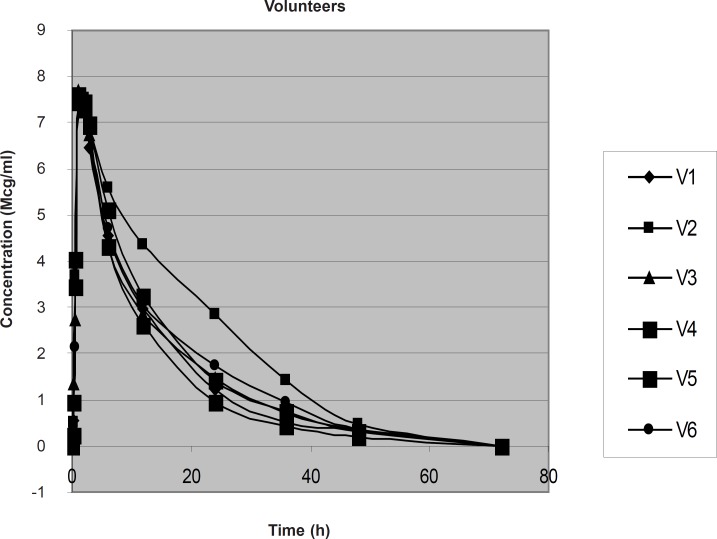
Plasma Conc. vs. Time graph of LF in 6 healthy volunteers

The concentration of drug in plasma of typhoid patients and in healthy volunteers at different time intervals was measured as shown in Table 2, Figure 2 and Table 3, Figure 3, respectively. 

**Table 2:** Mean ± SD Plasma Concentration (μg/mL) of LF at different time intervals following the oral administration of 500 mg to typhoid patients

**Time (h)**	**Mean**	**± SD**
0.25	0.5037	0.13
0.5	3.1313	0.38
1	7.4605	0.16
2	7.3625	0.16
3	6.7858	0.94
6	4.6249	0.192
12	2.8888	0.166
24	1.3925	0.154
36	0.7365	0.125
48	0.2169	0.071

**Table 3:** Mean ± SD Plasma Concentration (μg/mL) of LF at different time intervals following the oral administration of 500 mg to healthy volunteers

**Time (h)**	**Mean**	± **SD**
0.25	0.6667	0.42
0.5	3.3290	0.67
1	7.5939	0.7
2	7.4533	0.1
3	6.8109	0.2
6	4.7700	0.45
12	3.1820	0.55
24	1.6129	0.6
36	0.7939	0.33
48	0.3326	0.08


**Figure 4:**
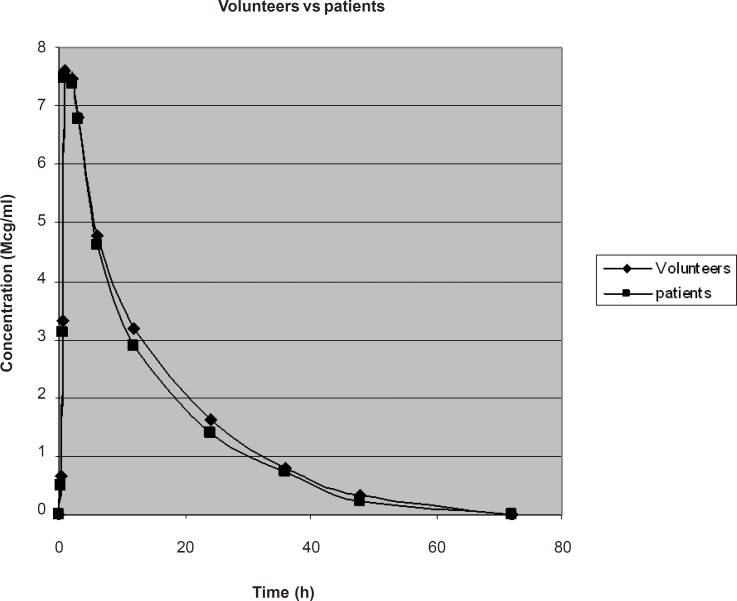
Graphical presentation of Average Plasma Conc. of LF in 6 healthy volunteers and in 6 typhoid patients

The comparison of average plasma concentration of LF in healthy volunteers and typhoid patients is shown in Figure 4. 

**Figure 5:**
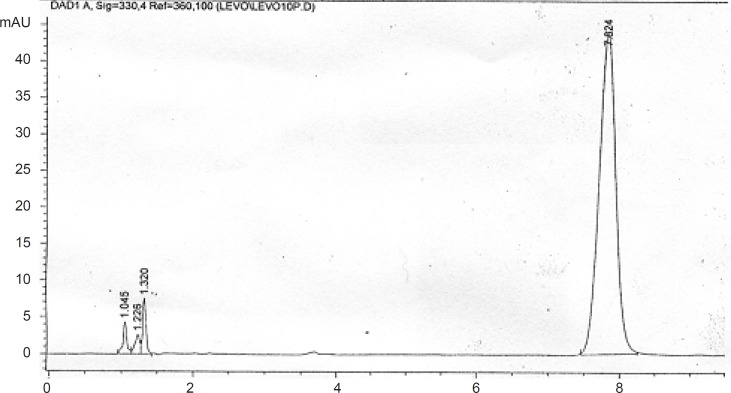
Chromatogram of LF 10 μg/mL in human plasma

The plasma concentration-time data was analyzed by two compartmental open models and the values of different pharmacokinetic parameter were determined in typhoid patients and in healthy volunteers. The comparison of average values of pharmacokinetic parameters in healthy volunteers and in typhoid patients is shown in Table 4. The difference between pharmacokinetic parameters of LF in healthy human volunteers and typhoid patients was calculated by using unpaired t-test. The p-values of AUC, Cl, Vd, T^1/2^, C_max_ and T_max_ were calculated as 0.40, 0.53, 0.22, 0.12, 0.68 and 0.86, respectively, which shows no difference of pharmacokinetics of LF in healthy volunteers and typhoid patients.

## Discussion

LF and other fluoroquinolones are rapidly cleared in Cystic fibrosis due to the enzyme induction and rapid renal clearance, therefore, doses higher than normal is required for achieving the therapeutic outcomes (13). Pharmacokinetic parameters of LF are calculated from plasma drug level measured by HPLC methods, through one compartment model and two compartment open models with first order elimination (14, 15). In one study, the LF concentration in plasma was measured at 330 nm UV range using C18 reverse phase column and methyl t-butyl ether for single-step liquid-liquid extraction and pharmacokinetic parameters from plasma drug concentration were calculated by using both one-compartment and two-compartment open model approaches (14). In healthy human volunteers, oral bioavailability of LF is 100%, C_max_ is 5.2 mg/L in plasma 1 to 2 h after the administration of 500 mg oral doses. V_D_ is 1.1 L/Kg, t_1/2_ is 6-8 h, 24-38% of drug bound to plasma proteins and approximately 80% of the drug is excreted through kidney both by glomerular filtration and active tubular secretion in chemically unchanged form. Metabolites of LF are pharmacologically inactive and only small fraction of it under goes hepatic metabolism. Although LF is rapidly distributed into tissues, penetration to cerebrospinal fluid is very poor (15). 

Different analytical techniques have been used for the determination of LF concentration in plasma. In this project, the concentration of LF was measured by standard HPLC method (16) which was standardized before the start of actual experiment.

The concentration of LF at different time intervals was determined in both typhoid patients and healthy volunteers. Two compartmental open models were selected to explain the pharmacokinetic parameters of LF in typhoid patients and in healthy volunteers. Different pharmacokinetic parameters of LF calculated by two-compartment model are discussed below separately.

*Area under curve AUC (mgh/L)*

Area under Curve AUC (mgh/L) is the total area under plasma concentration time curve from t_0_ to t_∞_. AUC of LF after 500 mg of oral dose was found to be 106.23 ± 8.72 mgh/L in healthy volunteers and 102.38 ± 4.63 mgh/L in typhoid patients. The difference is non-significant due to the absence of any effect of disease on the absorption of drug after the oral administration. This result corresponds to the area under curve 93 ± 31 mgh/L observed by Kiser *et al., *2005 (17). This difference in result may be due to the different environmental conditions.

*Total body clearance cl (l/h)*

Total body clearance in case of LF after 500 mg of oral dose was found to be 4.75 ± 0.42 L/h in healthy volunteers and 4.89 ± 0.22 L/h in typhoid patients. However, the result is much less than total body clearance of 9.0 ± 3.2 L/h observed by Kiser *et al., *2005 (17) which might be due to racial or environmental difference.

*Volume of distribution Vd (L)*

When LF was given as 500 mg of single oral dose, the average volume of distribution calculated by area method was 76.357 ± 8.06 L in healthy volunteers and 70.433 ± 6.13 L in typhoid patients. This result correlates with the volume of distribution observed by Kiser *et al., *2005 (17) which is 104.10 ± 12.48 L.

*Elimination half life t½ (h)*

The average elimination half life of LF after 500 mg of oral dose was found to be 11.148 ± 0.91 h in healthy volunteers and 10.018 ± 1.166 h in typhoid patients. This result is insignificant, which means that typhoid fever do not affect the elimination of LF. This is also closed to the elimination half life (9.31 ± 1.63 h) observed by Goodwin *et al., *1994 (18), but varies from the half life observed by Kiser *et al., *2005 (17) which was 7.8 ± 1.6 h that might be racial or environmental difference.

*Time to peak concentration T*_max_ *(h)*

In case of LF after 500 mg of oral dose, T_max_ was found to be 1.8645 h in healthy volunteers and 1.8191 h in typhoid patients with average ± SD of 0.2 and 0.1 in healthy volunteers and patients, respectively. The result correlates with T_max_ observed by Albarellos *et al., *2005 (19) which was 1.62 ± 0.84.

*Peak plasma concentration C*_max_ *(μg/mL)*

The C_max_ of LF after 500 mg of oral dose was found to be 7.5691 ± 0.2 μg/mL in healthy volunteers and 7.5991 ± 0.33 μg/mL in typhoid patients. There is no significant difference of C_max_ in typhoid patients and in healthy volunteers which shows that the absorption of LF after the oral administration is not affected by typhoid fever. This result is also closed to the C_max_ of 8.13 ± 1.64 mg/L determined by Benko *et al., *2007 (12).

## Conclusion

From all the observed data, no significant difference was found between the Pharmacokinetics of LF in healthy volunteers and in typhoid patients. It can be concluded that the typhoid fever has no effect on Pharmacokinetics of LF after the oral administration. Therefore, our recommendations are that the dose adjustment is not required for the administration of LF in typhoid patients.
